# Attenphos: General Phosphorylation Site Prediction Model Based on Attention Mechanism

**DOI:** 10.3390/ijms25031526

**Published:** 2024-01-26

**Authors:** Tao Song, Qing Yang, Peng Qu, Lian Qiao, Xun Wang

**Affiliations:** Qingdao Institute of Software, College of Computer Science and Technology, China University of Petroleum, Qingdao 266555, China; tsong@upc.edu.cn (T.S.); s21070069@s.upc.edu.cn (Q.Y.); s21070009@s.upc.edu.cn (P.Q.); s21070055@s.upc.edu.cn (L.Q.)

**Keywords:** protein phosphorylation, attention mechanism, phosphorylation site prediction

## Abstract

Phosphorylation site prediction has important application value in the field of bioinformatics. It can act as an important reference and help with protein function research, protein structure research, and drug discovery. So, it is of great significance to propose scientific and effective calculation methods to accurately predict phosphorylation sites. In this study, we propose a new method, Attenphos, based on the self-attention mechanism for predicting general phosphorylation sites in proteins. The method not only captures the long-range dependence information of proteins but also better represents the correlation between amino acids through feature vector encoding transformation. Attenphos takes advantage of the one-dimensional convolutional layer to reduce the number of model parameters, improve model efficiency and prediction accuracy, and enhance model generalization. Comparisons between our method and existing state-of-the-art prediction tools were made using balanced datasets from human proteins and unbalanced datasets from mouse proteins. We performed prediction comparisons using independent test sets. The results showed that Attenphos demonstrated the best overall performance in the prediction of Serine (S), Threonine (T), and Tyrosine (Y) sites on both balanced and unbalanced datasets. Compared to current state-of-the-art methods, Attenphos has significantly higher prediction accuracy. This proves the potential of Attenphos in accelerating the identification and functional analysis of protein phosphorylation sites and provides new tools and ideas for biological research and drug discovery.

## 1. Introduction

Protein is an important living organism component, regulating life activities, controlling growth and energy metabolism, etc. Post-translational modifications (PTMs) are important cellular control mechanisms that increase the functional diversity of the proteome through the covalent addition of functional groups or proteins, the hydrolytic cleavage of regulatory subunits, or the degradation of whole proteins [1]. Protein phosphorylation is the most studied PTM, and the most studied phosphorylation to date is on serine (S), threonine (T), and tyrosine (Y). Phosphorylation site prediction can help researchers understand protein function and interactions, particularly in the areas of signal transduction and metabolic pathways [2]. For example, it can help researchers to study the relationship between proteins and biological processes such as cell cycle, apoptosis, and cell proliferation. And phosphorylation site prediction can provide important information for protein structure studies, as phosphorylation can change the conformation and charge of proteins and can help researchers predict protein secondary structures, tertiary structures, and interactions [3]. In addition, some studies have shown that more than 30% of eukaryotic proteins can be phosphorylated, and most of these proteins are closely associated with the development of human diseases, such as cancer [4]. Therefore, the study of the principles, types, and mechanisms of protein phosphorylation is important for understanding the pathogenesis of human diseases. Research on protein phosphorylation site prediction can also help to identify new drug targets, thus providing new ideas and directions for disease treatment.

Early researchers performed many bioassay identification methods to identify phosphorylation sites, such as low-throughput 32P labeling [5] and high-throughput mass spectrometry [6], but such methods are quite time-consuming and laborious. With the rapid development of natural language processing, artificial intelligence, and other disciplines, and the integration of various new technologies with bioinformatics, the difficulties of traditional experimental methods have been effectively solved. Dozens of different methods, including machine learning, statistics, feature extraction, and other techniques [7,8,9], have been used to predict and identify phosphorylation sites. For example, Musite, proposed in 2010, used multiple features and algorithms to improve prediction accuracy by using local amino acid sequence frequencies, k-nearest neighbor features, and protein disorder scores. This was followed by MusiteDeep [10], the first deep learning framework for predicting general and kinase-specific phosphorylation sites, which was proposed by the team in 2017, using a convolutional neural network with a novel two-dimensional attention mechanism for prediction. In 2019, Fenglin Luo et al. proposed DeepPhos [11], which utilizes densely connected convolutional neuron network blocks to capture multiple representations of sequences. Final phosphorylation prediction was performed through intra-block crosstalk layers and inter-block crosstalk layers. These deep learning architectures for phosphorylation site prediction are far superior to traditional experimental methods. However, there is still room for improvement in prediction accuracy and prediction efficiency. It is essential to develop a prediction tool that performs well at S, T, and Y sites.

These deep learning architectures for phosphorylation site prediction are far superior to traditional experimental methods. However, there are more negative samples than positive samples for almost all PTM types, including protein phosphorylation [10], which makes the performance of these methods limited. By training with unbalanced datasets, the model can have better generalization ability in real situations. In addition, there is still a lot of room for improvement in the generalization ability of these models, which are not yet able to achieve a high level of prediction performance on S, T, and Y sites with small sample sizes at the same time. Therefore, there is still room for further improvement in terms of prediction accuracy and efficiency. Therefore, it has become crucial to develop a prediction tool that can perform well on both balanced and unbalanced datasets with higher accuracy and better generalization ability.

In response to the above challenges, this study proposes a new deep learning framework to accurately predict general phosphorylation sites using protein sequence information with good robustness and generalization ability. Unlike previous deep learning prediction methods, Attenphos consists of self-attention and CNN blocks that use three structurally identical modules with different window sizes to capture significant sequence representations of protein phosphorylation sites. The feature vector output of the attention module is converted using the Encoding conversion module, increasing the representation capability of the neural network. The transformed coded feature matrix has more structural information and learns features more fully than if the output features were processed directly. The final three outputs are further integrated to make the final prediction. To evaluate the performance of Attenphos, we collected a large number of validated phosphorylation examples for model training and evaluation, and we conducted experimental comparisons with some other prediction tools and deep learning methods. The experimental results show that our proposed method outperforms some existing prediction tools on both balanced and unbalanced datasets. Attenphos achieves the best overall prediction performance on T, S, and Y sites with small sample sizes.

## 2. Results

### 2.1. Comparison Experiments on Balanced Datasets

In this section, we provide a discussion of the comparative performance of Attenphos for general phosphorylation site prediction using a balanced dataset from human protein phosphorylation. We compared Attenphos with several existing general phosphorylation site prediction tools, including GPS 2.1 [12], NetPhos 3.0 [13], PPRED [14], Musite [15], PhosphoSVM [16], SKIPHOS [17], Deepphos [11], and Transphos [18]. We performed 10-fold cross-validation to train the model using the training datasets of the S, T, and Y sites. We tested it using an independent PPA test set. As shown in Table 1, the results indicate that our method obtains the best overall performance in S and T bit prediction. And on the Y dataset with a smaller sample size, our method still outperforms existing state-of-the-art deep learning architectures to obtain the best overall performance.

Specifically, at the S site, our model obtains the highest Mcc value of 0.443 and the highest Auc value of 0.788, while other metrics such as Sn, Sp, and F1 values are also in the lead. In the case of the Mcc metric, we achieve an improvement of 11% compared to the state-of-the-art prediction tool Transphos. At the T site, our method shows the best performance, with Attenphos obtaining the highest Mcc and Auc values compared to other methods. Compared to GPS 2.1, NetPhos 3.0, PPRED, Musite, PhosphoSVM, SKIPHOS, Deepphos, and Transphos, our architecture improves the Auc values by 2.1%, 5.1%, 11.3%, 6.9%, 2.6%, 4.8%, 1.7%, and 1.9%, respectively, and obtains the highest Mcc value (0.254). At the Y site, our method obtains the Auc value of 0.625, second only to the Auc value of SKIPHOS (0.634), and the highest Mcc value (0.201). Compared to state-of-the-art deep learning prediction tools such as Deepphos and Transphos, Attenphos improves a lot on Auc metrics, especially on Mcc metrics. To summarize, our model Attenphos performs well in phosphorylation prediction at the S, T, and Y sites, showing the best overall results, with Attenphos being able to accurately predict phosphorylation sites compared to existing state-of-the-art tools.

### 2.2. Comparison Experiments on Unbalanced Datasets

**Figure 1:**
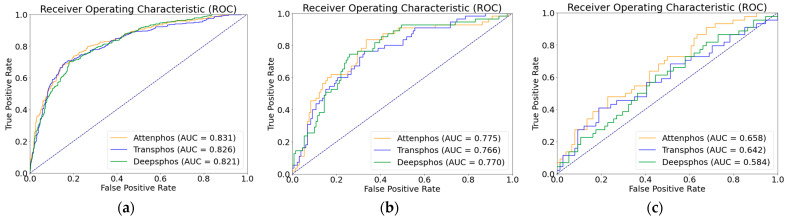
ROC curve plots of Attenphos, Transphos, and Deepphos on the mouse dataset ((**a**–**c**) indicate the S, T, and Y sites).

As shown in Table 2, we conducted performance comparison experiments on a dataset with positive and negative sample unbalance distribution, which is a phosphorylation dataset from mouse proteins. In order to assess the generalization capability of our model on unbalanced datasets with limited samples, we specifically selected the advanced deep learning models Transphos and Deepphos for a focused comparison. Both Transphos and Deepphos perform well on the balanced dataset. We trained and validated individual models, including Deepphos, Transphos, and Attenphos. Then, we tested them using independent test sets. Considering the unbalance in the number of positive and negative samples, we used evaluation metrics of SN, SP, MCC, AUC, and F1 scores for comparison. The results are shown in the table above. At the T site, Attenphos achieves the highest SP, AUC, MCC, SN, and F1 values of 87.79%, 0.775, 0.416, 50.91%, and 0.566, respectively. Compared with Transphos, its AUC value is increased by 0.9% and its MCC value by 2.2%. At the Y site, Attenphos achieves the highest SP, SN, AUC, F1, and MCC values. Attenphos obtains the highest MCC and F1 values in S-site phosphorylation prediction. Compared to Transphos, Attenphos shows an increase of 0.5% in the AUC value and 0.3% in the F1 score value. In addition, the MCC, SN, and SP values also perform better. Our research findings unveil the superiority of Attenpphos over Deepphos and Transphos, as confirmed by the ROC curve analysis. The ROC curves of Attenpphos consistently exhibit a larger area under the curve (AUC), indicating the better classification performance of our model compared to the other two models. The ROC graph is shown in Figure 1 below. This indicates that our model can still show excellent performance in the presence of positive and negative sample unbalance.

In real-world protein phosphorylation problems, where the distributions of positive and negative samples are often unbalanced, our model demonstrates significant performance improvement in phosphorylation prediction at the S, T, and Y sites, achieving higher overall performance. This suggests that Attenphos is not only applicable to datasets with balanced distributions but also to datasets with unbalanced distributions that are close to the real situation.

### 2.3. Ablation Experiments

**Figure 2:**
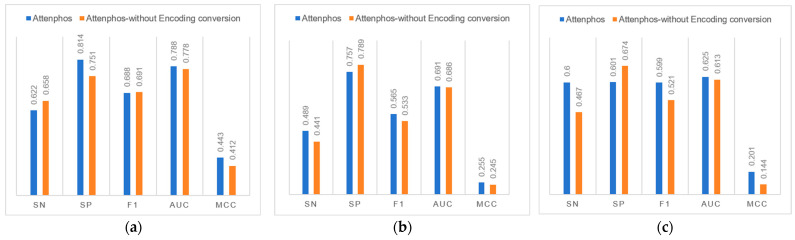
Comparison of ablation experiment results. (**a**), (**b**), and (**c**) indicate the comparison of experimental results at the S site, T site, and Y site, respectively.

We investigated the contribution of the Encoding conversion block to general phosphorylation site prediction. Figure 2 shows that removing the Encoding conversion block in the Attenphos architecture decreases the overall performance of general phosphorylation site prediction. For example, at the S site, removing the Encoding conversion causes the Sp, F1, Auc, and Mcc values to be reduced at the S site. At the T site, the removal of Encoding conversion reduces the values of Sn, F1, Auc, and Mcc. At the Y site, the removal of Encoding conversion reduces the values of Sn, F1, Auc, and Mcc. Therefore, the use of the Encoding conversion module allows the model to better capture the relational features prior to the amino acids, focusing on key information and thus improving the overall predictive performance of the model.

In this section, we performed ablation experiments on the effect of the attention mechanism in the Attenphos method. The experiment results are shown in Table 3 below. As shown in the table, the performance of Attenphos-noAttention undergoes a large decrease compared to Attenphos at the S, T, and Y sites. Although the F1 score improves by 1% and 0.6% at the S and T sites, other metrics such as Mcc and Auc show a large decrease. At the Y site, Attenphos-noAttention decreases by 6.1% in AUC, 7.2% in mcc, and 5.1% in F1 score. Self-attention mechanisms can focus on the relationship between different amino acids. The self-attention mechanism module enables our model to better and more efficiently capture the long-range dependencies of protein sequences and extract global feature information, thus improving the prediction accuracy.

### 2.4. Feature Visualization

**Figure 3:**
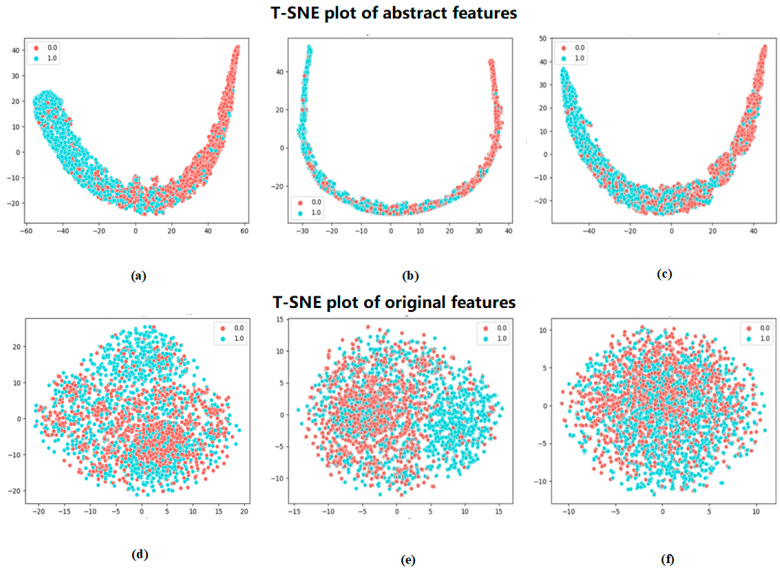
t-SNE visualization of Attenphos extracted features and embedding encoded features. (**a**), (**b**), and (**c**) represent the visualization plots after feature extraction using Attenphos at the S, T, and Y sites, respectively, and (**d**), (**e**), and (**f**) represent the feature visualization plots after encoding using embedding at the S, T, and Y sites, respectively.

To more visually demonstrate the effectiveness of the method in this paper for the classification of phosphorylated versus non-phosphorylated sites, the features extracted by Attenphos were visualized. We chose a commonly used dimensionality reduction visualization method, t-SNE (t-Distributed Stochastic Neighbor Embedding) [19], which can be used to map high-dimensional data into a low-dimensional space and preserve the relative distance relationship between the data. It can reduce the dimensionality of the high-dimensional features and visualize the results. Figure 3 shows the visualization of the original features encoded by embedding and the features extracted by Attenphos. It is clear that the features encoded by embedding are visualized in a cluttered manner. However, the extracted features of Attenphos can easily separate phosphorylated and unphosphorylated samples, indicating that our method can predict the generalized phosphorylated site with high accuracy.

## 3. Discussion

We propose a new deep learning method named Attenphos. It can predict potential general phosphorylation sites. And experiments comparing our model with existing methods show that our method has better overall performance compared to other existing methods. The protein sequences are centered on the prediction sites, intercepting short sequences with different window sizes. Short sequences are encoded and input into three modules with the same structure, in which the output features of the self-attention block are transformed and encoded. Then, when convolution is used, effective features can be better extracted. Finally, the three parts are combined into a combined feature to fully improve the generalization ability of the enhanced model and the prediction of the model accuracy. And self-attention can effectively solve the problem of long-distance dependence on protein phosphorylation.

Although our method has achieved better performance, there are still some shortcomings for improvement. (1) In future work, we can improve the framework using some deep learning modules that work well for classification, such as BiGRU [20], which can be explored in the next work. (2) We could attempt to include information other than the protein sequence. For example, protein structure information can provide information about the environment of amino acid residues, such as residue accessibility, local secondary structure, inter-residue distance, etc. PPI [21] information can provide association information in protein interaction networks, such as the tendency of proteins in the same signaling pathway to share phosphorylation sites. So, we could try to combine protein sequence, structure, and PPI information and use deep learning models to perform prediction, which can be further explored in future work. (3) Due to the small kinase-specific phosphorylation dataset available and the small number of samples, our model does not have good prediction performance on small samples, and more advanced model architecture is needed for kinase phosphorylation prediction, which will be the goal of our next work.

In summary, our deep learning method Attenphos shows superior performance in protein general phosphorylation site prediction with high accuracy and stability. The method is based on an attention mechanism which works by filtering out the most predictive features and optimal model parameters for prediction. Our method can provide a useful tool not only in basic research but also in biomedical and drug discovery applications, helping to accelerate the identification and study of phosphorylation sites. This study provides a strong reference for future bioinformatics and proteomics studies and offers new ideas for understanding the mechanisms of protein phosphorylation regulation.

## 4. Material and Methods

### 4.1. Datasets

This study is based on the prediction of general phosphorylation sites. We used the phosphorylation site training dataset provided by Phospho.ELM [22], a database containing experimentally validated, manually curated phosphorylation sites. By integrating information from literature reports and experimental data, the Phospho.ELM database provides extensive annotations of phosphorylation sites and related biological information. We used human protein phosphorylation data and mouse protein phosphorylation data from this database.

For mouse phosphorylation data, as shown in Table 4, our study used the mouse protein phosphorylation dataset from the PELM database and removed redundant data using the CD-HIT tool [23] with a redundancy threshold set at 40%. Since the number of positive and negative samples for phosphorylation is not balanced in the real world, we chose negative samples in this study at a ratio of 1:2 for positive and negative samples. The negative samples were randomly selected on the same protein sequence, excluding identical amino acids outside the labeled phosphorylation sites. Firstly, the dataset was randomly divided into an 80% training set and 20% independent test set, and secondly, 10% of the training set was used as a validation set during training to prevent overfitting.

For phosphorylation data from human proteins, we used the same training dataset, validation set, and independent test set as in Transphos [18]. The number of positive and negative samples in the dataset was balanced. Among them, the training dataset was obtained from the human phosphorylation data in the Phospho.ELM database, as shown in Table 5. Meanwhile, the independent test set was obtained from the PhosPhAt (PPA) [24,25,26] database, as shown in Table 6. In the above way, we constructed the phosphorylation site datasets for training and evaluation to support our research work.

In order to better extract the features near the phosphorylation site, we separately cut the original sequence into short sequences of different lengths centered on the prediction site for experiments. We explored many window combinations by conducting experiments and finally chose the optimal input window combination of 21, 33, and 51. We chose sequence fragments with left and right lengths of 10, 16, and 25, centered on the prediction site. If the left and right lengths of the predicted site were insufficient, * was filled in to ensure that each subsequence had the same length, and the negative samples were manipulated as above. We digitally encoded the short protein sequences. Then, the encoded sequence digital representation was encoded using embedding. When encoding with one-hot, it cannot represent the relationship between adjacent amino acids very well, meaning that the efficiency of training is not very high. The use of embedding can optimize this problem very well. The feature vectors after embedding encoding were 21 × 16, 33 × 16, and 51 × 16.

### 4.2. Model Architecture

**Figure 4:**
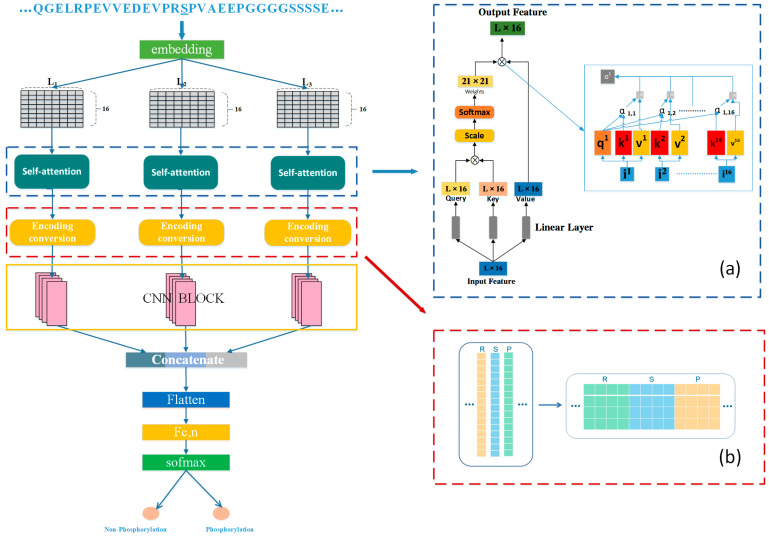
Attenphos framework. (**a**) represents the self-attention mechanism, and (**b**) represents encoding conversion.

The deep learning architecture of Attenphos is shown in Figure 4. We first processed the protein sequence length and extracted short sequences with window sizes of 21, 33, and 51. Experiments have shown that the length combinations of 21, 33, and 51 are the most appropriate. Then, we encoded short sequences by embedding and input the encoded feature vectors to three structurally identical self-attention blocks for feature extraction. Using self-attention could fully obtain the long-range dependence information of protein sequences. Then, the output features were separately transformed and encoded using the Encoding conversion module, converting each feature of length 16 into 4 × 4 features to better learn the relationship features between amino acids. Then, the transformed feature vectors were input into a one-dimensional convolutional block. The resulting three-part output vectors were combined into an integrated feature. Then, it was input to a flatten layer, transformed into a one-dimensional tensor. And then, it passed through several layers of fully connected layers. Finally, the output results were obtained by softmax.

#### 4.2.1. Self-Attention

For neural networks, the input vectors are generally of different sizes. There is a certain relationship between different vectors, which cannot be reflected by the embedding vectors. But the actual training cannot give full play to the relationship among these inputs, resulting in very poor model training results.

The self-attention mechanism is introduced in this model, the specific structure of which is shown in Figure 1a, and the self-attention mechanism is one of the attention mechanisms. The problem of poor model performance due to the inability to establish a correlation between the relevant inputs above can be solved by this. This allows our model to notice correlations between different parts of the whole input and to learn the importance of the features, providing more weight to the more important features to represent the sequence.

In the self-attention mechanism, the input sequence is first subjected to feature extraction. It usually uses a linear transformation to map the input sequence into the Query, Key, and Value vector spaces. Specifically, for the input sequence *X* = {*x*1, *x*2, …, *x*n}, Query, Key, and Value can be generated in the following ways:

(1) $$Q=XW_{Q}$$

(2) $$K=XW_{k}$$

(3) $$V=XW_{V}$$

where *X* denotes the input sequence;$W_{Q}$, $W_{k}$, and $W_{V}$ is the learnable weight matrix; and *Q*, *K*, and *V* denote the generated Query, Key, and Value, respectively.

(4) $$s_{ij}=q_{i}\cdot k_{j}$$

(5) $$\alpha_{ij}=\frac{\exp(s_{ij}/\sqrt{d_{k}})}{\sum_{j=1}^{n}\exp\left(\frac{s_{ij}}{\sqrt{d_{k}}}\right)}$$

where $q_{i}$ denotes the *i*-th element of the Query vector, $k_{j}$ denotes the *j*-th element of the Key vector, $d_{k}$ denotes the dimension of the Key vector, and $\alpha_{ij}$ denotes the attention weight between position *i* and position *j*.

(6) $$o_{i}=\sum_{j=1}^{n}\alpha_{ij}v_{j}$$

where $v_{j}$ denotes the *j*-th element of the Value vector and $o_{i}$ denotes the *i*-th element of the Output vector.

In our model, we used a self-attention layer with 16 input units and output features of the win (window length) ×16, which allowed the correlation between the features of each amino acid to be better expressed.

#### 4.2.2. Encoding Conversion

**Figure 5:**
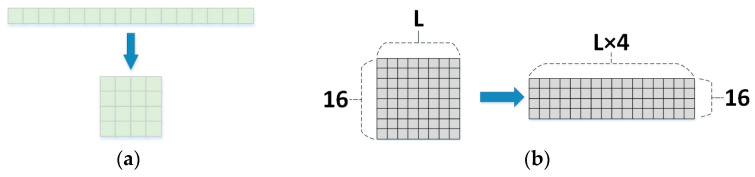
(**a**) Conversion example. (**b**) Encoding conversion module converted feature representation.

We transformed the feature vector after the output of self-attention by changing the features of length 16 into 4 × 4 features, as shown in Figure 4b and Figure 5a,b. This is because turning the 16-length features into 4 × 4 features increased the representation capability of the neural network. The 4 × 4 feature matrix had more structural information. This approach can better take advantage of the one-dimensional convolutional layer when processing sequential data to improve the model efficiency and the generalization capability of the model, and it also can reduce the number of model parameters. For example, if the original data are considered as image data, a higher dimensional feature matrix retains more detailed information in the image and helps the network to better learn the features in the image. This transformation encoding the features in a way that allows for better learning of amino acid sequences. The transformed feature vectors were fed into a one-dimensional convolutional layer with the stride set to 4 and the convolutional kernel set to 8. This configuration allowed the relationships between amino acids to be fully learned and more detailed information to be retained. We verified through experimental results that this method helped in the extraction of model features and improved the accuracy of the model prediction.

#### 4.2.3. Model Training

Attenphos is a deep learning framework designed to predict sites in protein sequences where phosphorylation is likely to occur. The framework uses a self-attention mechanism to learn the interactions between amino acid residues to extract effective feature vectors for phosphorylation site prediction. To prevent overfitting, Attenphos uses dropout layers in the convolutional and fully connected layers. The model uses the categorical cross-entropy loss function, which is minimized by the Adam algorithm, and L2 regularization, which adjusts the model parameters to better fit the data in training. The specific hyper-parameter choices are shown in Table 7.

(7) $${Loss}_{c}=-\frac{1}{N}\sum_{j=1}^{N}y^{i}\ln P(y^{i}=1|{\;x}^{j})+(1-y^{i})\ln P(y^{i}=0|\;x^{j})$$

*N* is the number of samples, $x^{j}$ refers to the *j*th input localized sequence, and $y^{j}$ refers to the corresponding phosphorylated state label of the *j*th input sequence.

#### 4.2.4. Metrics

Several commonly used measurements were used to assess the performance of Attenphos, including the area under the ROC curve (AUC), sensitivity (*Sn*), specificity (*Sp*), F1 score, and the Matthew correlation coefficient (Mcc). The calculation formulas are shown below:

(8) $$Sn=\frac{TP}{TP+FN}$$

(9) $$Sp=\frac{TN}{TN+FP}$$

(10) $$F1=\frac{2\times Pre\times Sn}{Pre+Sn}$$

(11) $$Mcc=\frac{TP\times TN-FP\times FN}{\sqrt{\left(TP+FN\right)\times \left(TP+FP\right)\times \left(TN+FN\right)\times \left(TN+FP\right)}}$$

*TP* (true positive): a prediction of 1 and a true value of 1, i.e., true positive; *FP* (false positive): a prediction of 1 and a true value of 0, i.e., false positive; *TN* (true negative): a prediction of 0 and a true value of 0, i.e., true negative; *FN* (false negative): a prediction of 0 and a true value of 1, i.e., false negative.

## 5. Conclusions

In this study, we propose a new framework, Attenphos, based on deep learning to accurately predict general phosphorylation sites using protein sequence information. Attenphos has good robustness and generalization ability. Unlike previous deep learning prediction methods, Attenphos consists of self-attention and CNN modules that use three modules with the same structure and different window sizes to capture important sequence representations of protein phosphorylation sites. On the balanced dataset, Attenphos achieved AUC values of 0.788, 0.691, and 0.625 at the S, T, and Y sites, respectively. On the unbalanced dataset for mouse species, AUC values of 0.831, 0.775, and 0.658 and MCC values of 0.511, 0.416, and 0.262 were achieved at the S, T, and Y sites, respectively. It was demonstrated that Attenphos achieved better results on both balanced and unbalanced datasets. The experiment proves that our model outperforms other existing methods and deep learning models in all aspects. Also, we experimentally proved the importance of the Encoding conversion block. Adding the Encoding conversion block can significantly improve the performance of the model. We chose a commonly used downscaling visualization method, t-SNE, and the features extracted by our method, Attenphos, can easily separate phosphorylated sites from unphosphorylated sites compared to the features encoded by embedding.

## Figures and Tables

**Table 1:** Model comparison on balanced dataset. (**a**), (**b**), and (**c**) represent the results of model comparison for S site, T site, and Y site, respectively.

(**a**)					
Methods	Sn(%)	Sp(%)	AUC	Mcc	F1
GPS 2.1	22.20	95.26	0.670	0.135	0.350
NetPhos 3.0	28.55	87.23	0.643	0.081	0.404
PPRED	21.32	94.00	0.676	0.107	0.335
Musite	28.60	95.21	0.726	0.182	0.429
PhosphoSVM	34.01	**95.90**	0.776	0.237	0.492
SKIPHOS	46.20	68.60	0.691	0.265	0.520
Deepphos	66.43	75.89	0.775	0.425	0.697
transphos	**67.16**	75.89	0.787	0.432	**0.702**
Attenphos	62.15	81.36	**0.788**	**0.443**	0.688
(**b**)					
Methods	Sn(%)	Sp(%)	AUC	Mcc	F1
GPS 2.1	22.20	95.26	0.670	0.135	0.350
NetPhos 3.0	28.55	87.23	0.640	0.081	0.404
PPRED	26.43	83.51	0.578	0.052	0.370
Musite	15.56	**95.36**	0.622	0.098	0.259
PhosphoSVM	21.79	93.41	0.665	0.115	0.339
SKIPHOS	**65.80**	58.60	0.643	0.197	**0.635**
Deepphos	46.02	76.04	0.674	0.231	0.542
transphos	47.32	76.22	0.672	0.246	0.553
Attenphos	48.93	75.68	**0.691**	**0.254**	0.565
(**c**)					
Methods	Sn(%)	Sp(%)	AUC	Mcc	F1
GPS 2.1	47.93	60.83	0.552	0.043	0.512
NetPhos 3.0	63.91	46.10	0.554	0.048	0.587
PPRED	42.01	65.08	0.539	0.064	0.475
Musite	28.85	81.71	0.587	0.064	0.392
PhosphoSVM	28.55	**84.39**	0.595	0.084	0.396
SKIPHOS	**65.80**	58.60	**0.634**	0.197	**0.635**
Deepphos	49.93	66.37	0.621	0.165	0.545
transphos	38.52	72.30	0.601	0.115	0.463
Attenphos	59.56	60.59	0.625	**0.201**	0.599

Data in bold indicates that the model performs best for that evaluation metric.

**Table 2:** Performance comparison on unbalanced mouse dataset. (**a**), (**b**), and (**c**) denote the comparison of the prediction results of Attenphos with the state-of-the-art deep learning prediction tools at the S site, T site, and Y site, respectively.

(**a**)					
Methods	SP(%)	SN(%)	AUC	MCC	F1
Attenphos	84.01	**66.98**	**0.831**	0.511	**0.672**
Transphos	**85.41**	65.08	0.826	**0.512**	0.669
Deepphos	84.64	61.27	0.821	0.469	0.637
(**b**)					
Methods	SP(%)	SN(%)	AUC	MCC	F1
Attenphos	87.79	**50.91**	**0.775**	**0.416**	**0.566**
Transphos	86.26	**50.91**	0.766	0.393	0.554
Deepphos	**89.31**	30.91	0.77	0.248	0.395
(**c**)					
Methods	SP(%)	SN(%)	AUC	MCC	F1
Attenphos	**75.68**	**50.00**	**0.658**	**0.262**	**0.524**
Transphos	72.00	**50.00**	0.642	0.221	0.506
Deepphos	67.57	38.64	0.584	0.126	0.400

Data in bold indicates that the model performs best for that evaluation metric. For each column, deeper colors mean that the model performs better on that metric.

**Table 3:** Ablation experiments of attention mechanisms in the Attenphos method. (**a**), (**b**), and (**c**) indicate the S, T, and Y sites.

(**a**)					
Methods	Sn(%)	Sp(%)	AUC	Mcc	F1
Attenphos	62.15	**81.36**	**0.788**	**0.443**	0.688
Attenphos-noAttention	**67.59**	73.79	0.707	0.415	**0.698**
(**b**)					
Methods	Sn(%)	Sp(%)	AUC	Mcc	F1
Attenphos	48.93	**75.68**	**0.691**	**0.254**	0.565
Attenphos-noAttention	**52.65**	68.25	0.604	0.211	**0.571**
(**c**)					
Methods	Sn(%)	Sp(%)	AUC	Mcc	F1
Attenphos	**59.56**	**60.59**	**0.625**	**0.201**	**0.599**
Attenphos-noAttention	52.74	60.15	0.564	0.129	0.548
Deepphos	67.57	38.64	0.584	0.126	0.400

Data in bold indicates that the model performs best for that evaluation metric.

**Table 4:** PELM. Mouse protein phosphorylation dataset.

Site	Positive	Negative
S	1589	3178
T	310	620
Y	201	402

**Table 5:** PELM. Human protein phosphorylation training set.

Site	Positive	Negative
S	20,968	20,968
T	5685	5685
Y	2163	2163

**Table 6:** PPA test dataset.

Site	Positive	Negative
S	5437	5437
T	1686	1686
Y	676	676

**Table 7:** Model parameter values.

Parameters Settings	Parameters Settings
Epochs	100
Learning rate	0.0001
Dropout	0.3
EMBEDDING DIM	16
DENSE	256, 84, 2
Batch size	32

## Data Availability

The Attenphos project’s source code and datasets are publicly available at https://github.com/uuy99/Attenphos, accessed on 18 January 2024.

## References

[1] Mann M., Jensen O.N. (2003). Proteomic analysis of post-translational modifications. Nat. Biotechnol..

[2] Cohen P.T. (2002). Protein phosphatase 1–targeted in many directions. J. Cell Sci..

[3] Groban E.S., Narayanan A., Jacobson M.P. (2006). Conformational changes in protein loops and helices induced by post-translational phosphorylation. PLoS Comput. Biol..

[4] Li F., Li C., Marquez-Lago T.T., Leier A., Akutsu T., Purcell A.W., Smith A.I., Lithgow T., Daly R.J., Song J. (2018). Quokka: A comprehensive tool for rapid and accurate prediction of kinase family-specific phosphorylation sites in the human proteome. Bioinformatics.

[5] Aponte A.M., Phillips D., Harris R.A., Blinova K., French S., Johnson D.T., Balaban R.S. (2009). 32P labeling of protein phosphorylation and metabolite association in the mitochondria matrix. Methods Enzymol..

[6] Beausoleil S.A., Villén J., Gerber S.A., Rush J., Gygi S.P. (2006). A probability-based approach for high-throughput protein phosphorylation analysis and site localization. Nat. Biotechnol..

[7] Trost B., Maleki F., Kusalik A., Napper S. (2016). DAPPLE 2: A tool for the homology-based prediction of post-translational modification sites. J. Proteome Res..

[8] Qin G.-M., Li R.-Y., Zhao X.-M. (2017). PhosD: Inferring kinase–substrate interactions based on protein domains. Bioinformatics.

[9] Huang S.-Y., Shi S.-P., Qiu J.-D., Liu M.-C. (2015). Using support vector machines to identify protein phosphorylation sites in viruses. J. Mol. Graph. Model..

[10] Wang D., Zeng S., Xu C., Qiu W., Liang Y., Joshi T., Xu D. (2017). MusiteDeep: A deep-learning framework for general and kinase-specific phosphorylation site prediction. Bioinformatics.

[11] Luo F., Wang M., Liu Y., Zhao X.-M., Li A. (2019). DeepPhos: Prediction of protein phosphorylation sites with deep learning. Bioinformatics.

[12] Xue Y., Ren J., Gao X., Jin C., Wen L., Yao X. (2008). GPS 2.0, a tool to predict kinase-specific phosphorylation sites in hierarchy. Mol. Cell. Proteom..

[13] Blom N., Gammeltoft S., Brunak S. (1999). Sequence and structure-based prediction of eukaryotic protein phosphorylation sites. J. Mol. Biol..

[14] Basu S., Plewczynski D. (2010). AMS 3.0: Prediction of post-translational modifications. BMC Bioinform..

[15] Gao J., Thelen J.J., Dunker A.K., Xu D. (2010). Musite, a tool for global prediction of general and kinase-specific phosphorylation sites. Mol. Cell. Proteom..

[16] Dou Y., Yao B., Zhang C. (2014). PhosphoSVM: Prediction of phosphorylation sites by integrating various protein sequence attributes with a support vector machine. Amino Acids.

[17] Dang T.H., Trac Q.T., Phan H.K., Nguyen M.C., Thi Q.T.P. (2019). SKIPHOS: Non-kinase specific phosphorylation site prediction with random forests and amino acid skip-gram embeddings. BioRxiv.

[18] Wang X., Zhang Z., Zhang C., Meng X., Shi X., Qu P. (2022). Transphos: A deep-learning model for general phosphorylation site prediction based on transformer-encoder architecture. Int. J. Mol. Sci..

[19] Van der Maaten L., Hinton G. (2008). Visualizing data using t-SNE. J. Mach. Learn. Res..

[20] Lin X., Quan Z., Wang Z.-J., Huang H., Zeng X. (2020). A novel molecular representation with BiGRU neural networks for learning atom. Brief. Bioinform..

[21] Jones S., Thornton J.M. (1996). Principles of protein-protein interactions. Proc. Natl. Acad. Sci. USA.

[22] Dinkel H., Chica C., Via A., Gould C.M., Jensen L.J., Gibson T.J., Diella F. (2010). Phospho.ELM: A database of phosphorylation sites—Update 2011. Nucleic Acids Res..

[23] Fu L., Niu B., Zhu Z., Wu S., Li W. (2012). CD-HIT: Accelerated for clustering the next-generation sequencing data. Bioinformatics.

[24] Durek P., Schmidt R., Heazlewood J.L., Jones A., MacLean D., Nagel A., Kersten B., Schulze W.X. (2010). PhosPhAt: The Arabidopsis thaliana phosphorylation site database. An update. Nucleic Acids Res..

[25] Heazlewood J.L., Durek P., Hummel J., Selbig J., Weckwerth W., Walther D., Schulze W.X. (2007). PhosPhAt: A database of phosphorylation sites in Arabidopsis thaliana and a plant-specific phosphorylation site predictor. Nucleic Acids Res..

[26] Zulawski M., Braginets R., Schulze W.X. (2012). PhosPhAt goes kinases—Searchable protein kinase target information in the plant phosphorylation site database PhosPhAt. Nucleic Acids Res..

